# Identification and Characterization of the HicAB Toxin-Antitoxin System in the Opportunistic Pathogen *Pseudomonas aeruginosa*

**DOI:** 10.3390/toxins8040113

**Published:** 2016-04-19

**Authors:** Gang Li, Mengyu Shen, Shuguang Lu, Shuai Le, Yinling Tan, Jing Wang, Xia Zhao, Wei Shen, Keke Guo, Yuhui Yang, Hongbin Zhu, Xiancai Rao, Fuquan Hu, Ming Li

**Affiliations:** Department of Microbiology, Third Military Medical University, Chongqing 400038, China; liganglg1990@163.com (G.L.); shenmengyu890905@163.com (M.S.); shulang88@126.com (S.L.); leshuai2004@tmmu.edu.cn (S.L.); tanyinling2011@163.com (Y.T.); wangjing2008@aliyun.com (J.W.); zhaoxia413@163.com (X.Z.); shenwei356@gmail.com (W.S.); doublekeguo@163.com (K.G.); yangyuhui199090@126.com (Y.Y.); zhulaobiao1991@163.com (H.Z.); raoxiancai@126.com (X.R.)

**Keywords:** toxin-antitoxin system, HicAB, *Pseudomonas aeruginosa*, biofilm formation, virulence

## Abstract

Toxin-antitoxin (TA) systems are small genetic modules that are widely distributed in the genomes of bacteria and archaea and have been proposed to fulfill numerous functions. Here, we describe the identification and characterization of a type II TA system, comprising the *hicAB* locus in the human opportunistic pathogen *Pseudomonas aeruginosa*. The *hicAB* locus consists of genes *hicA* and *hicB* encoding a toxin and its cognate antitoxin, respectively. BLAST analysis revealed that *hicAB* is prevalent in approximately 36% of *P. aeruginosa* strains and locates in the same genomic region. RT-PCR demonstrated that *hicAB* forms a bicistronic operon that is cotranscribed under normal growth conditions. Overproduction of HicA inhibited the growth of *Escherichia coli*, and this effect could be counteracted by co-expression of HicB. The *Escherichia coli* kill/rescue assay showed that the effect of HicA is bacteriostatic, rather than bactericidal. Deletion of *hicAB* had no effect on the biofilm formation and virulence of *P. aeruginosa* in a mice infection model. Collectively, this study presents the first characterization of the HicAB system in the opportunistic pathogen *P. aeruginosa*.

## 1. Introduction

Toxin-antitoxin (TA) systems are abundant, diverse and small genetic modules that are widely distributed in the genomes of bacteria and archaea [1,2]. TA systems, typically consisting of a stable toxin and a labile antitoxin, were initially identified on plasmids contributing to plasmid stabilization through genetic addiction [3,4]. To date, numerous plasmid- and chromosome-encoded TA systems have been identified and characterized. According to the nature and mode of action of the antitoxins, TA systems have been grouped into five types [5]. In type I and III TA systems, the antitoxins are small RNAs that either inhibit the synthesis of the toxin (type I) or sequester it (type III) [6]. Types IV and V are two recently-described TA systems, in which the proteinic antitoxins either act as antagonists (type IV) or inhibit the translation of their cognate toxin (type V) [5]. Type II TA systems, in which both toxin and antitoxin are proteins, are the most documented and highly abundant in the genomes of bacteria, and the toxicity of toxin is inhibited by forming the antitoxin-toxin complex directly [7].

In the increasingly high-throughput genome sequencing era, a large collection of sequence data become available in several databases. Analyses of genome sequences have revealed that TA systems are prevalent in nearly all bacterial species, including pathogens and non-pathogens [1,8]. Different types of TA systems and even multiple copies of the same type contained in a bacterial genome have also been reported, for example the chromosome of *Escherichia coli* and *Mycobacterium tuberculosis* possesses 36 and 79 TA loci, respectively [2]. Since the first discovery of the TA system as an addiction module, numerous biological functions have been proposed for TA systems, including plasmid stabilization, programmed cell death, stress responses, phage resistance, persister cell formation, biofilm formation and pathogenicity [2,9,10]. High prevalence and various functions make TA systems attract persistent concern.

*Pseudomonas aeruginosa* inhabits diverse ecological niches, causes significant morbidity and mortality among immune-compromised individuals and resists treatment with antibiotics due mainly to its notable biofilm formation and multi-drug resistance [11]. To date, the first TA system termed HigB/HigA has been identified in this notorious opportunistic pathogen and linked to virulence [12]. Identification and characterization of other TA systems in *P. aeruginosa* will be beneficial to gain further insight into the biological characteristics and pathogenesis of this versatile opportunistic pathogen.

The *hicAB* locus belongs to one of the well-characterized type II TA systems. This locus was first described as an insertion into the major pilus gene cluster in several strains of *Haemophilus influenza* and subsequently was predicted to be a novel TA system using a comparative-genomic approach [13,14]. In *E. coli* K-12, the *hicAB* locus was first identified as an active TA system, in which ectopic production of toxin HicA induced cleavage in three model mRNAs (*ompA*, *dksA* and *rpoD* mRNAs) and tmRNA by a ribosome-independent manner, concomitantly reducing the global rate of translation, while HicB functions as an antitoxin and neutralizes HicA [14]. The crystal structure of the HicA3-HicB3 complex of *Yersinia pestis*, which belongs to the HicAB family, has been determined, showing that HicB3 forms a tetramer that can bind two HicA3 toxin molecules and occludes the HicA3 active site through its *N*-terminal domain [15]. Several functions have been proposed for HicAB, including persister cell formation and involvement in extracytoplasmic stress responses [16,17]. Hitherto, the *hicAB* locus has been found in numerous bacterial and archaeal genomes and characterized in several bacterial species [14,15,18,19,20,21], but data on the presence, prevalence, diversity and biological role of the HicAB system in *P. aeruginosa* still remain unknown.

In this study, the *hicAB* locus was identified in *P. aeruginosa* by homology search, and its prevalence was investigated. The results showed that *hicAB* forms a bicistronic operon that is cotranscribed under normal growth conditions and constitutes an active TA system. The HicAB system appears to be not involved in the biofilm formation and virulence of *P. aeruginosa*, and its biological role needs to be further elucidated. To our knowledge, this is the first characterization of the HicAB system in *P. aeruginosa*.

## 2. Results

### 2.1. Identification and Prevalence of the hicAB Locus in P. aeruginosa

**Figure 1:**
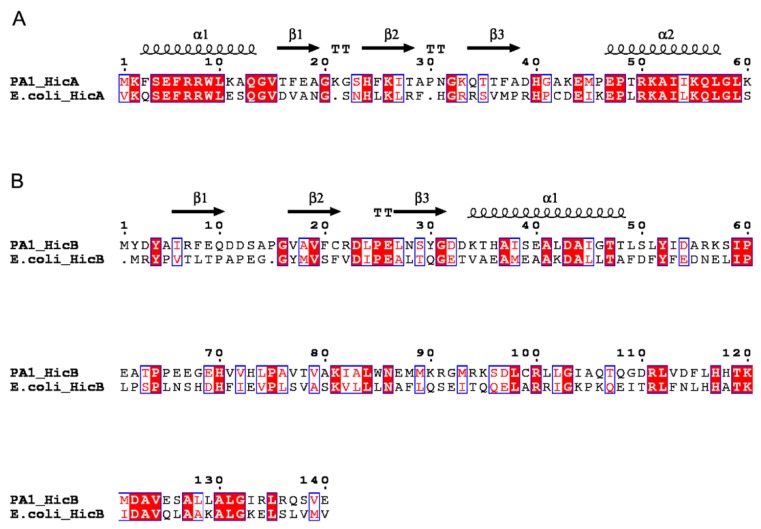
Sequence alignments of the *P. aeruginosa* HicAB system with related homologs. (**A**) Alignment of the HicA proteins. (**B**) Alignment of the HicB proteins. Identical residues are shown as white letters with red background, and similar residues are shown as red letters with white background. The predicted secondary structures of *P. aeruginosa* HicA and HicB are shown at the top. α: α-helix; β: β-sheet; T: turn.

In the genome of *P. aeruginosa* PA1, the gene *PA1S_06925* encodes a 60-aa protein annotated as a putative mRNA interferase [22]. BlastP analysis revealed that it shares 47% identity with the *E. coli* HicA toxin, thus termed *hicA*. The other gene, *PA1S_06920*, encoding a 140-aa protein that shares 30% identity with the *E. coli* HicB antitoxin, was named *hicB*. Protein homology modeling using CPHmodels predicted the structures of *P. aeruginosa* HicA and HicB based on the available solved three-dimensional structure of proteins TTHA1913 (PDB: 1WHZ) and TTHA1756 (PDB: 2YZT), respectively [23,24]. The secondary structure of HicA showed that it is likely to adopt an α1β1β2β3α2 fold characteristic of a double-stranded RNA (dsRNA)-binding domain (Figure 1A), and this fold is conserved in the HicA family. The histidine 24 (His24) residue of HicA may be functionally important, since it is conserved in *E. coli* HicA, *B. pseudomallei* HicA and *Y. pestis* HicA1 toxins and has been experimentally verified in some species [13,15,16]. HicB contains an α-helix and three β-sheets at its *N*-terminal domain, adopting a partially-degraded RNase H fold (Figure 1B). In addition, the helix-turn-helix (HTH) DNA-binding domain of the Xre family is fused to the HicB *N*-terminal domain, which is indicative of antitoxin involved in DNA binding.

To determine the prevalence of the *hicAB* locus in *P. aeruginosa*, a BlastN search was performed against the complete and draft genomes of *P. aeruginosa* available in the Pseudomonas Genome Database as of 10 March 2016 [25]. The results suggested that approximately 36% (363 out of 996) of *P. aeruginosa* strains harbor the *hicAB* locus, including *P. aeruginosa* LESB58 and other LES-like strains with high pathogenicity. The detailed information is listed in Table S1. Searching the vicinities of the *hicAB* locus in *P. aeruginosa* PA1 genome revealed that the downstream region encodes several proteins annotated as hypothetical proteins on the opposite strand, while interestingly, the upstream region on the opposite strand next to *hicA* encodes many proteins homologous to bacteriophage proteins, such as holin (PA1S_06930), glycoside hydrolase (PA1S_06935), terminase (PA1S_06950, PA1S_06955) and portal protein (PA1S_06960). These results indicate the horizontal gene transfer (HGT) of *hicAB*, which is consistent with previous analysis [13]. In all of the available complete genomes of *P. aeruginosa* that possess the *hicAB* locus, *hicAB* is linked to sequences encoding phage-related proteins and has the same genomic location as in PA1. In addition, HicA and HicB of *P. aeruginosa* PA1 share 100% and 78% amino acid identity with other homologues among these strains, respectively (Figure S1), suggesting that *hicAB* is conserved in *P. aeruginosa* and may be involved in a particular biological process.

### 2.2. Genetic Organization and Transcriptional Analysis of the hicAB Locus

**Figure 2:**
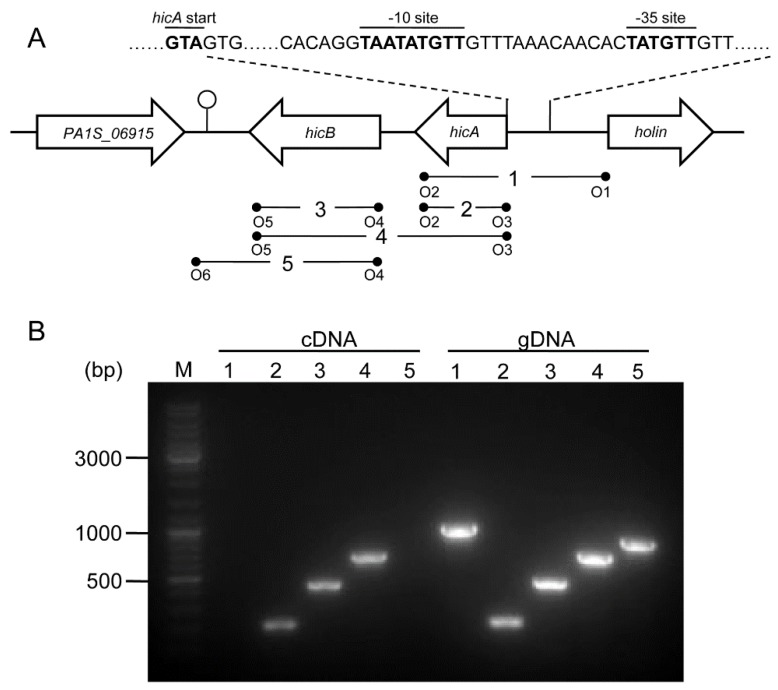
Genetic organization and transcriptional analysis of the *hicAB* locus. (**A**) Genetic organization of *hicAB* in *P. aeruginosa* PA1. The open arrows represent the location and orientation of the genes (not drawn to scale). The putative −35 and −10 sites located upstream of the *hicA* gene are indicated as bold letters. The stem-loop structure shows the potential transcriptional terminator located downstream of the *hicB* gene. The primer pairs used for RT-PCR or PCR are denoted below. (**B**) Co-transcription analysis of *hicAB*. cDNA and gDNA were amplified using primer pairs depicted in (A), respectively. The DNA marker is shown on the left (M).

A genetic organization analysis revealed that *hicA* is located upstream of *hicB*; the two genes are separated by 46 bp, and the adjacent genes are all encoded on the opposite strand, suggesting that the *hicA* and *hicB* genes are organized in a bicistronic operon (Figure 2A). BPROM (Bacterial sigma70 promoter prediction program) analysis of the upstream region of the *hicA* gene identified a putative bacterial sigma70 promoter located 23 bp upstream of the *hicA* start code ATG, with the inferred −35 (TTGTAT) and −10 (TTGTATAAT) sites (Figure 2A). FindTerm analysis of the downstream region of the *hicB* gene revealed a putative rho-independent bacterial terminator. The inferred promoter and terminator suggest that *hicAB* comprises a transcriptional unit.

To characterize the coupling transcription between *hicA* and *hicB*, a reverse transcription polymerase chain reaction (RT-PCR) analysis was performed. cDNA was synthesized using cellular total RNA extracted from log-phase cultures and amplified using a set of primer pairs (Figure 2A); genomic DNA (gDNA) was amplified using the same primer pairs as the control. The results showed that the PCR products were of the expected sizes for *hicA*, *hicB* and *hicAB* and consistent with that of the gDNA (Figure 2B). However, when cDNA was used as the template, no PCR products of the flanking sequences of *hicAB* were detected, but as for the gDNA template, the cognate PCR products were positive (Figure 2B). These results indicated that *P. aeruginosa* *hicA* and *hicB* form a bicistronic operon and are actively co-transcribed under normal growth conditions.

### 2.3. Ectopic Production of HicA Induces Growth Arrest of E. coli, which Can Be Alleviated by HicB

**Figure 3:**
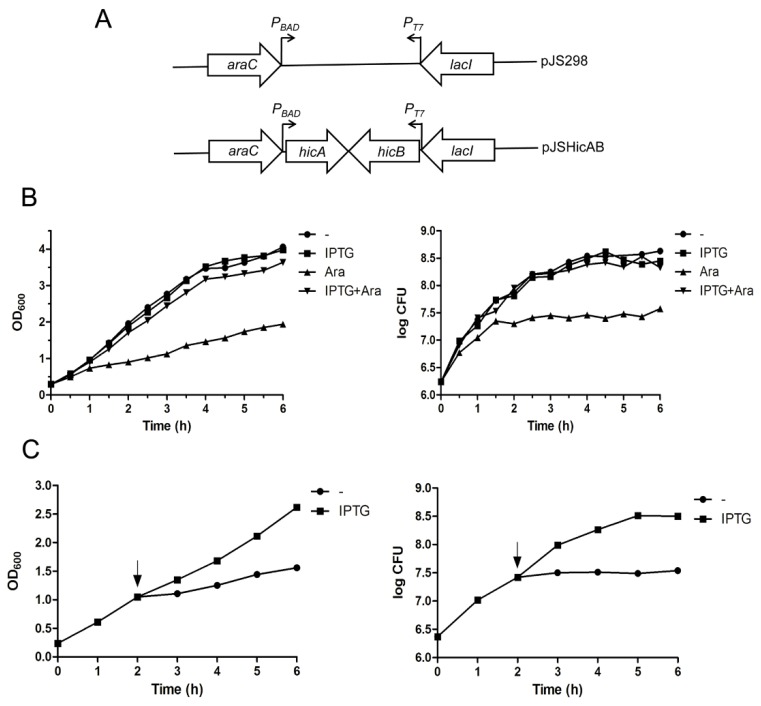
Effects of HicA and HicB overexpression on the growth of *E. coli*. (**A**) Schematic diagram of plasmids pJS298 and pJSHicAB designed for selective expression of *hicA* and *hicB* under the *P_BAD_* and *P_T7_* promoters, respectively; (**B**) Overproduction of HicA causes cell growth inhibition, which can be counteracted by HicB. *E. coli* BL21(DE3)/pLysS cells containing plasmid pJSHicAB were grown to an OD_600_ of ~0.3 and supplemented with the indicated inducers to express *hicA* and/or *hicB*. Bacterial growth was monitored by OD_600_ (left panel) and CFU (right panel) assessment; (**C**) Toxicity of HicA could be rescued by HicB that was induced subsequently. *E. coli* cells growing to an OD_600_ of ~0.3 were added with l-arabinose to induce HicA expression (at the time point of 0 h). Two hours later, IPTG was supplemented to induce HicB production (indicated as arrows). Bacterial growth was monitored by OD_600_ (left panel) and CFU (right panel) measurement. These data represent a typical profile of three independent experiments.

The crucial characteristic of TA systems is that the antitoxin can counteract the toxin-induced growth inhibition. To determine whether HicAB is indeed a functional TA system, a dual conditional expression system was constructed to investigate the effect of HicA and HicB on the growth of *E. coli* strain BL21(DE3)/pLysS. In this system, the *hicB* and *hicA* genes were cloned successively into the pJS298 expression vector [26]. Thus, the recombinant vector was named pJSHicAB, in which the arabinose-inducible promoter *P_BAD_* and IPTG-inducible promoter *P_T7_* control the expression of *hicA* and *hicB* on the same vector backbone in *trans*, respectively (Figure 3A).

In the bacterial growth assay, *E. coli* BL21(DE3)/pLysS cells harboring the pJSHicAB plasmid showed no difference in growth in the presence of IPTG that induced *hicB* gene expression alone, as indicated in Figure 3B. However, when the HicA toxin was induced alone by the addition of l-arabinose (Ara), the bacteria growth was inhibited (Figure 3B). Cell viability determination indicated that viable cell counts increased slightly for the first 1.5 h after induction of HicA, then were maintained at a stable level (Figure 3B, right panel). In contrast, cell growth inhibition in the OD_600_ value and cell viable counts was not observed when *hicA* and *hicB* were co-expressed by the addition of l-arabinose and IPTG simultaneously (Figure 3B). These results suggested that overproduction of HicA is toxic and causes cell growth arrest, which can be alleviated by HicB, even when the *hicB* gene was provided in *trans*.

### 2.4. Overexpression of HicA Confers Cell Stasis, which Can Be Rescued by Subsequently Induced HicB

To further determine whether overproduction of HicA confers cell death or cell stasis, we examined the effect of subsequent production of HicB on the HicA-induced cell growth inhibition. As seen in Figure 3C, the OD_600_ value and CFU of *E. coli* cells increased immediately upon HicB induction by the addition of IPTG, indicating that the toxicity of HicA could be rescued by the action of HicB that was induced subsequently. Taken together, we concluded that the effect of HicA in *E. coli* is bacteriostatic rather than bactericidal.

**Figure 4:**
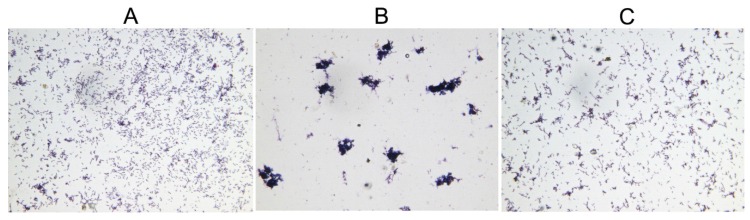
HicA overproduction causes aggregation of *E. coli* cells, which can be reverted by HicB induced subsequently. (**A**) Control; (**B**) Induction of HicA; (**C**) Subsequent induction of HicB. Microscopic images of *E. coli* cells were captured digitally (Gram staining, ×400).

Overproduction of toxins has been proposed to induce bacterial morphological changes, including cell filamentation, spherical cell formation, lemon-shaped cell formation and ghost cell formation [5,27,28,29,30]. During the growth assay, microscopic examination of the *E. coli* BL21(DE3)/pLysS cells showed that the normal-sized cells became aggregated after induction of HicA, and this phenotype can be reverted by subsequent induced HicB (Figure 4). This toxin-induced phenomenon has not been documented previously, and the underlying mechanism needs to be further elucidated.

### 2.5. Deletion of hicAB Has No Effect on the Biofilm Formation and Virulence of P. aeruginosa

To investigate the possible biological role of the *hicAB* locus in *P. aeruginosa*, an isogenic *hicAB* knockout mutant of *P. aeruginosa* PA1, termed PA1∆HicAB, was constructed through homologous recombination. DNA sequencing results also confirmed successful allelic replacement of the *hicAB* locus by the gentamicin resistance cassette (Gm).

**Figure 5:**
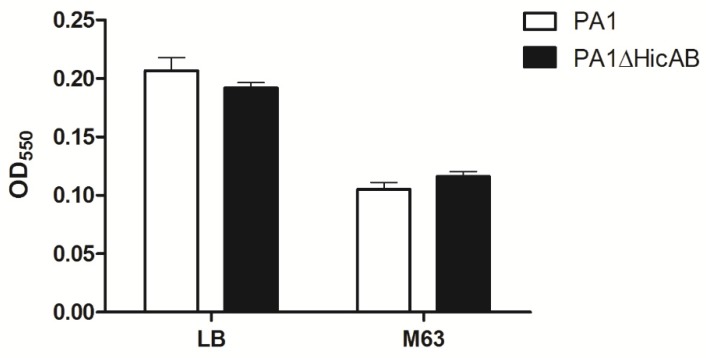
Biofilm formation of PA1 and PA1∆HicAB in LB and M63 broth. Biofilm was formed at 37 °C for 24 h, washed and stained with crystal violet, then the OD_550_ was measured. No statistically-significant difference between PA1 and PA1∆HicAB was observed in either media (*p* > 0.05). Data are expressed as the mean ± SEM of three independent experiments.

To determine whether *hicAB* of *P. aeruginosa* is involved in biofilm formation, a microtiter dish assay was performed. The results showed that there were no significant difference of biofilm formation between PA1 and PA1∆HicAB, either in the case of LB or M63 broth (a standard biofilm assay medium for *P. aeruginosa*) (Figure 5). To assess the role of *hicAB* in the pathogenicity of *P. aeruginosa*, we performed an experimental infection model in BALB/c female mice. Groups of ten mice were inoculated intraperitoneally with 3 × 10^7^ CFU of PA1 and PA1∆HicAB individually, and the survival rate was measured. After 24 h, the survival rates of mice in PA1 and PA1∆HicAB were 20% and 10%, respectively, and no significant difference was observed between the two groups, suggesting that *hicAB* may not be involved in *P. aeruginosa* virulence.

## 3. Discussion

Due to its ubiquitous distribution in bacterial genomes and various functions, TA systems have attracted increasing concern in recent years. To date, TA systems have been identified in numerous bacterial genomes, with many being well characterized. Unfortunately, the related knowledge of the notorious opportunistic pathogen *P. aeruginosa* was rarely documented. This study identified and characterized the first HicAB system (a type II TA system) in *P. aeruginosa*. Based on the predicted identity of the amino acid sequence and their secondary structure, transcriptional analysis and the cell growth assay, we demonstrated that the *hicAB* locus has the properties required for a typical TA system belonging to the HicAB family [13,15]. Analyzing the action of HicA in *E. coli* K-12 suggested that HicA functions as a ribosome-independent mRNA interferase that blocks translation by cleaving mRNAs and causes cell growth inhibition [14]. As the homolog of *E. coli* HicA, we therefore reasoned that the *P. aeruginosa* HicA induces cell growth arrest via a similar mechanism.

Previous studies of the distribution of the *hicAB* locus in numerous bacterial genomes showed that *hicAB* is transferred horizontally [13]. Consistent with this mode of transmission, several *hicAB* cassettes were found to be encoded in prophages and plasmids that could serve as the vehicles for HGT. Moreover, no virtual collinearity in the localization of *hicAB* was found in the genomes of closely-related species. In this study, we found that the flanking regions of *hicAB* encode several proteins homologous to phage-related proteins, including holin and terminase. Thus, we deduced that *hicAB* of *P. aeruginosa* appears to be a mobile element acquired through HGT. Furthermore, analysis of the *hicAB* locus in different *P. aeruginosa* strains revealed that *hicAB* locates in a conserved genomic region, implying that *hicAB* may be involved in a particular biological process that remains unknown and needs to be further characterized.

*E. coli* has been usually used as the host for verification of heterogenic TA components due mainly to its well-established conditional expression systems [31,32,33]. In the growth assay of *E. coli* BL21(DE3)/pLysS cells, HicA functions as a toxin and leads to cell stasis; meanwhile, HicB could rescue the bacteria cells from growth inhibition by co- or subsequent induction, indicating that the toxicity of HicA is bacteriostatic and can be alleviated by HicB. These results are consistent with previous reports of HicAB in several bacterial species [14,15,19]. An interesting finding during the cell growth was that the normal-shaped *E. coli* cells became aggregated after induction of HicA, and subsequently induced HicB could revert this phenotype. The underlying mechanism is still mysterious. Cell aggregation has been proposed to be an important adaptive strategy ensuring the survival and growth of bacteria in adverse environments [34,35]. Thus, we inferred that aggregation induced by HicA may be an adaptation adopted by *E. coli* cells to cope with the stress triggered by toxin overproduction.

To investigate the possible biological role of *hicAB* in *P. aeruginosa*, a *hicAB* knockout mutant was constructed. No significant difference between the WT and the *hicAB* deletion mutant was observed in terms of their biofilm formation and virulence in a mice infection model. Indeed, most TA deletion mutants show no discernible phenotype [2], which is partly due to functional redundancy and cross-talking of TA systems [36,37]. For example, progressive deletions of all ten TA systems in *E. coli* induced a cumulative effect on persister cell formation [36], and cross-talking between homologous or non-homologous TA systems has been documented in *M. tuberculosis* [38,39]. Another possible reason for TA deletion mutants exhibiting no deleterious phenotype may be due to the poor knowledge of the stresses activating TA systems. For example, some TA systems are activated only by a specific stress, thus becoming dispensable under normal growth conditions [40].

*P. aeruginosa* is a notorious opportunistic pathogen and resists lots of antibiotics due mainly to its notable biofilm formation and intrinsic drug resistance; thus, *P. aeruginosa*-associated infections are always intractable challenges in the clinical setting. Based on the wide spread of TA systems in bacterial genomes and the ability of toxins to regulate bacterial cell growth, the exploitation of TA systems as a novel antibacterial strategy via artificial activation of toxin has been proposed and has considerable potential [41,42,43]. Therefore, the understanding of the cellular targets, activation and biological roles of *P. aeruginosa* TA systems (including HicAB) might be greatly beneficial to develop novel and effective strategies to control this versatile opportunistic pathogen.

## 4. Materials and Methods

### 4.1. Bacterial Strains, Plasmids and Growth Conditions

The bacterial strains and plasmids used in this study are listed in Table 1. Both *P. aeruginosa* and *E. coli* strains were cultured in LB broth at 37 °C with shaking at 200 rpm or plated on LB agar, unless otherwise specified. When necessary, antibiotics were added at the following concentrations: for *E. coli*, gentamicin, 15 μg/mL; kanamycin, 50 μg/mL; for *P. aeruginosa*, gentamicin, 100 μg/mL.

### 4.2. Blast Alignment and Bioinformatics Analysis

Sequence homology searches and conserved domain analyses were carried out using the Basic Local Alignment Search Tool (BLAST) on the NCBI website (http://www.ncbi.nlm.nih.gov/BLAST) and on the Pseudomonas Genome Database (http://www.pseudomonas.com/) [25]. Multiple protein sequences were aligned using ClustalW (http://www.ebi.ac.uk/Tools/msa/clustalw2/) [45], and the figure was generated with ESPript (http://espript.ibcp.fr/ESPript/cgi-bin/ESPript.cgi) [46]. The modeled structures of *P. aeruginosa* HicA and HicB were generated using the CPHmodels 3.2 Server (http://www.cbs.dtu.dk/services/CPHmodels) [23]. The putative promoter and terminator were predicted by BPROM (http://linux1.softberry.com/berry.phtml) and FindTerm (http://www.softberry.com/berry.phtml?topic=findterm&group=programs&subgroup=gfindb), respectively.

### 4.3. DNA Extraction, RNA Purification and RT-PCR

Genomic DNA (gDNA) was extracted from overnight cultures of *P. aeruginosa* using the TIANamp Bacteria DNA Kit (Tiangen, Beijing, China) according to the manufacturer’s instructions. Total RNA was isolated from log-phase cultures using the Tripure Isolation Reagent (Roche, Basel, Basel-Stadt, Switzerland) following the manufacturer’s protocol. cDNA was synthesized from total RNA using random primers with RevertAid First Strand cDNA Synthesis Kit (Thermo Scientific, Waltham, MA, USA) according to the manufacturer’s recommendations. gDNA and cDNA were used as templates for PCR amplification and coupling transcriptional analysis of the *hicAB* locus and the flanking sequences.

### 4.4. E. coli Growth Assay

To determine if the *hicAB* locus encodes an active TA system, the conditional expression plasmid pJSHicAB was constructed to expression of *hicA* and *hicB* under the l-arabinose-inducible *P_BAD_* promoter and the IPTG-inducible *P_T7_* promoter, respectively. Briefly, the *hicB* gene was amplified from PA1 genomic DNA using primers HicB-T7F and HicB-T7R (Table S2), digested with *Nde* I and *Nco* I (FastDigest, Thermo Scientific, Waltham, MA, USA) and cloned under the *P_T7_* promoter into pJS298, resulting in pJSHicB. The *hicA* gene was amplified using primers HicA-BADF and HicA-BADR and cloned behind the *P_BAD_* promoter of pJSHicB using *Sac* I and *Nco* I restriction sites, generating the recombinant plasmid pJSHicAB.

In growth assays, *E. coli* BL21(DE3)/pLysS cells harboring the plasmid pJSHicAB were cultured in 100 mL of LB broth supplemented with 50 μg/mL kanamycin and 0.2% glucose to an OD_600_ of ~0.3. For selective expression of *hicA* and *hicB* genes, the culture was divided into four equal parts, of which three were provided with 1 mM of IPTG, 0.2% l-arabinose (Ara) and both (Sangon Biotech, Shanghai, China), respectively. The cultures were continued at 37 °C for 6 h, and aliquots were removed every 30 min for measurement of OD_600_ and enumeration of CFU. In the kill/rescue assay, *E. coli* culture was supplemented with 0.2% l-arabinose at an OD_600_ of ~0.3 and continued for 2 h. Then, the culture was divided into two equal parts, of which one was supplemented with 1 mM of IPTG. The cultures were continued for 4 h, and the OD_600_ and CFU were assessed every 1 h.

Microscopic examination of the *E. coli* cells was performed as described previously [27]. Briefly, *E. coli* BL21(DE3)/pLysS cells harboring the plasmid pJSHicAB were grown to an OD_600_ of 0.3, then induced with and without HicA expression and continued for 4 h. Gram staining of the cultures was performed; at the same time, the HicA overproduction culture was induced with HicB expression and continued for 2 h. Gram staining was done again to test whether HicB could revert the aggregated phenotype. Microscopic images were captured digitally (×400).

### 4.5. Construction of hicAB Deletion Mutant

The *hicAB* deletion mutant of PA1 was constructed through homologous recombination. Briefly, the left flanking region (~1000 bp) and right flanking region (~1000 bp) of the *hicAB* locus were amplified using primer pairs of LA-F/LA-R and RA-F/RA-R, respectively. The gentamicin resistance cassette was amplified from plasmid pUCP24 using primers Gm-OF and Gm-OR. The fragment of LA + Gm + RA was obtained using overlap PCR and cloned into plasmid pEX18Tc using *Bam*H I and *Hin*d III restriction sites, resulting in plasmid pEX∆HicAB. Then, pEX∆HicAB was electroporated into PA1 as described previously [47]. The *hicAB* deletion mutant was screened on LB agar supplemented with 100 μg/mL gentamicin and confirmed by PCR detection and sequencing.

### 4.6. Biofilm Formation Assay

The biofilm formation assay was performed as described previously [48]. Briefly, overnight cultures of PA1 and PA1∆HicAB were diluted 1:100 into fresh LB and M63 medium, respectively. Biofilm was formed at 37 °C for 24 h in a 96-well dish (Corning, New York, NY, USA) with 4 replicate wells for each treatment, washed and stained with crystal violet (Sangon Biotech, Shanghai, China). OD_550_ was quantified to represent biofilm formation.

### 4.7. Mouse Infection Experiment

The mouse infection experiment was performed as described previously [44]. Bacteria were grown in LB broth until the mid-log phase. Cells were collected, washed and resuspended in saline to a final concentration of 3 × 10^7^ CFU. Each strain (1 mL) was injected intraperitoneally into 6- to 8-week-old BALB/c female mice, and animals were monitored for 24 h. All the experiments were approved by the Laboratory Animal Welfare and Ethics Committee of the Third Mililary Medical University (Project identification code: SYXK-PLA-20120031, Approved at 15 December 2015).

### 4.8. Statistical Analysis

All statistical analyses were carried out using GraphPad Prism (Version 5.01, GraphPad Software Inc., La Jolla, CA, USA, 2007). Where appropriate, the data were analyzed using Student’s *t*-test. Differences were considered statistically significant at *p* < 0.05.

## Figures and Tables

**Table 1:** Bacterial strains and plasmids used in this study.

Strains/plasmids	Characteristics ^a^	Source/reference
Strains		
*P. aeruginosa* strains PA1	A multi-drug-resistant strain isolated from a patient with respiratory tract infection	Lab collection [22]
PA1∆HicAB	PA1 derivative with the *hicAB* locus replaced by a *Gm^r^* gene cassette; Gm^r^	This work
*E. coli* strains DH5α	Cloning host for maintaining recombinant plasmids	Tiangen
BL21(DE3)/pLysS	Expression host for exogenous protein production	Tiangen
Plasmids		
pJS298	A selective expression vector containing the *P_BAD_* and *P_T7_* promoters in *trans*; Kan^r^	[27]
pJSHicB	Derivative of pJS298 containing the *hicB* gene under the *P_T7_* promoter; Kan^r^	This work
pJSHicAB	Derivative of pJSHicB containing the *hicA* gene under the *P_BAD_* promoter; Kan^r^	This work
pUCP24	pUC18-based broad host-range vector; Gm^r^	Lab collection [44]
pEX18Tc	Gene replacement vector; Tet^r^	Lab collection [44]
pEX∆HicAB	Derivative of pEX18Tc designed for knockout of the *hicAB* locus; Gm^r^, Tet^r^	This work

^a^ Gm^r^, gentamicin resistant; Kan^r^, kanamycin resistant; Tet^r^, tetracycline resistant.

## Supplementary Materials

The following are available online at www.mdpi.com/2072-6651/8/4/113/s1: Figure S1: Genomic location and sequence alignment of the *hicAB* locus in selected *P. aeruginosa* genomes; Table S1: Prevalence of the *hicAB* locus in *P. aeruginosa* strains; Table S2: Primers used in this study.
