# Supplementary material for: Identification and Characterization of the HicAB Toxin-Antitoxin System in the Opportunistic Pathogen Pseudomonas aeruginosa
Source: Toxins (Basel). 2016 Apr 19;8(4):113. doi: 10.3390/toxins8040113 (PMC4848639; doi:10.3390/toxins8040113)
Supplement: Supplementary file 1 [file toxins-08-00113-s001.zip › toxins-125813-supplementary-for conversion.pdf]

Supplementary Materials: Identification and Characterization of the HicAB Toxin-Antitoxin System in the Opportunistic Pathogen *Pseudomonas aeruginosa*

Gang Li, Mengyu Shen, Shuguang Lu, Shuai Le, Yinling Tan, Jing Wang, Xia Zhao, Wei Shen, Keke Guo, Yuhui Yang, Hongbin Zhu, Xiancai Rao, Fuquan Hu *, Ming Li *

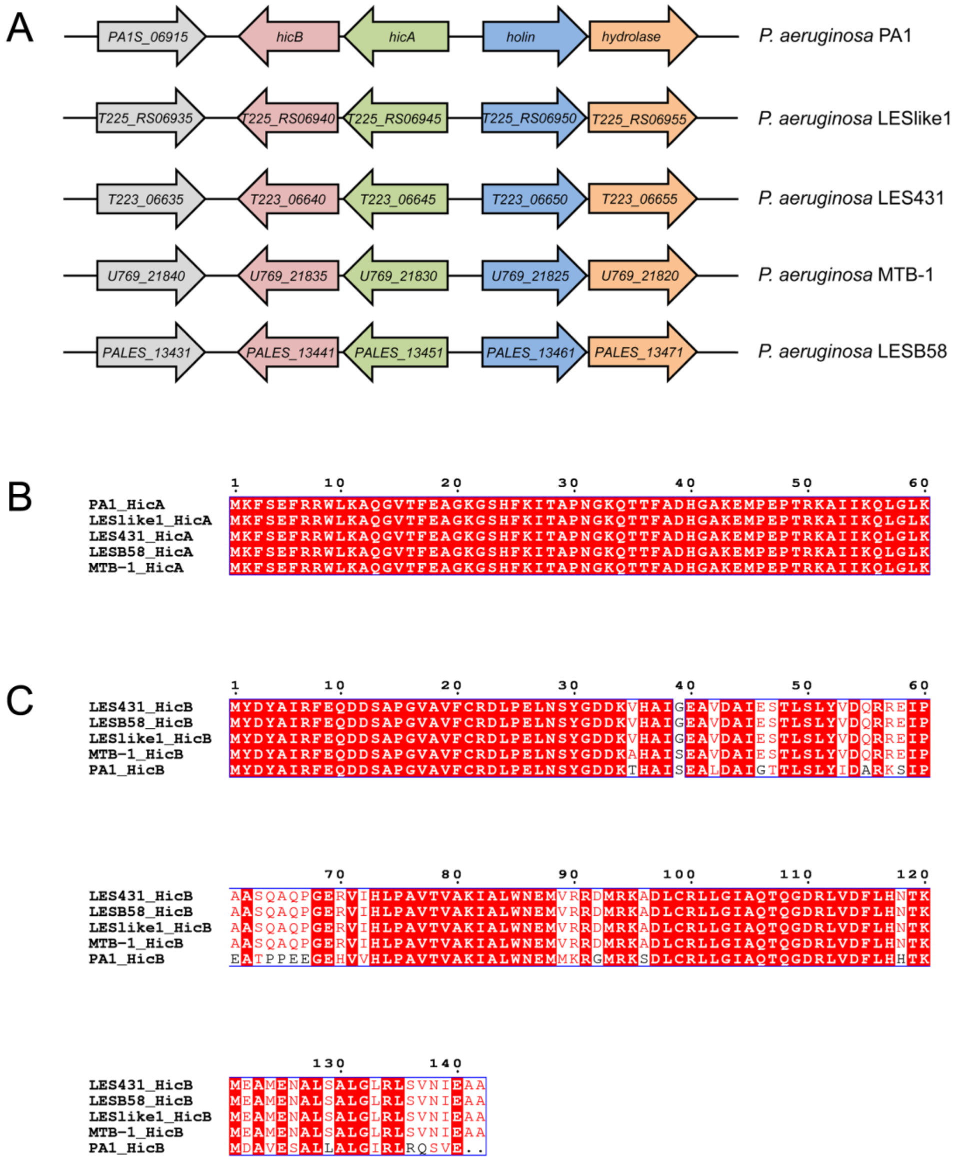

Figure S1. Genomic location and sequence alignment of the *hicAB* locus in selected *P. aeruginosa* genomes; (**A**) Genomic location of the *hicAB* locus in selected *P. aeruginosa* genomes. Arrows show a direction of transcription of the corresponding gene. Homologs are shown by the same color. Genes are not shown to scale; (**B**) Alignment of the HicA proteins; (**C**) Alignment of the HicB proteins. Identical residues are shown as white letters with red background, and similar residues are shown as red letters with white background. The GenBank accession numbers are as follows: *P. aeruginosa* PA1, CP004054.2; *P. aeruginosa* LESlike1, NZ_CP006984; *P. aeruginosa* LES431, NC_023066; *P. aeruginosa* MTB-1, NC_023019; *P. aeruginosa* LESB58, NC_011770.

Table S2. Primers used in this study.

Primers	Sequence(5′-3′)	Function
O1	GCCTTTCATCCTATCTCTGC	For transcription analysis
O2	TCATTTGAGCCCCAGTTGCTTG	For transcription analysis
O3	ATGAAGTTCAGCGAATTCAGACG	For transcription analysis
O4	ATGTACGACTATGCAATCCGTTTC	For transcription analysis
O5	TCACTCAACTGATTGACGAAGC	For transcription analysis
O6	GACTTGCTGTCATCACTCCATC	For transcription analysis
HicA-BAD	GCGAGCTCGAAGTTCAGCGAATTCAGACG (*Sac* I)	For *hicA* cloning into pJS298
HicA-BADR	TGCCATGGTCATTTGAGCCCCAGTTGCTTG (*Nco* I)	For *hicA* cloning into pJS298
HicB-T7F	GCCATATGTACGACTATGCAATCCGTTTC (*Nde* I)	For *hicB* cloning into pJS298
HicB-T7R	TGCCATGGTCACTCAACTGATTGACGAAGC (*Nco* I)	For *hicB* cloning into pJS298
LA-F	CGGGATCCTCGATTGCTGGGTGCATGCT (*Bam*H I)	For amplification of the left region of the *hicAB* locus
LA-R	GCTTATGTCAATTCGGTCAGGTTTGACGAGGTGAA	For amplification of the left region of the *hicAB* locus
Gm-OF	CTCGTCAAACCTGACCGAATTGACATAAGCCTGTT	For amplification of the Gm^r cassette
Gm-OR	AACAGAGACGAGGTGCGAATTGGCCGCGGCGTTGT	For amplification of the Gm^r cassette
RA-F	GCCGCGGCCAATTCGCACCTCGTCTCTGTTGTTTG	For amplification of the right region of the *hicAB* locus
RA-R	CCAAGCTTGCGGGGCGGAAATGAAAAAG (*Hin*d III)	For amplification of the right region of the *hicAB* locus
